# A Delphi Study to Identify Research Priorities Regarding Physical Activity, Sedentary Behavior and Sleep in Pregnancy

**DOI:** 10.3390/ijerph19052909

**Published:** 2022-03-02

**Authors:** Áine Brislane, Melanie J. Hayman, Margie H. Davenport

**Affiliations:** 1 Program for Pregnancy & Postpartum Health, Neurovascular Health Lab, Faculty of Kinesiology, Sport, and Recreation, Women and Children’s Health Research Institute, Alberta Diabetes Institute, University of Alberta, Edmonton, AB T6G 2E3, Canada; brislane@ualberta.ca; 2 Appleton Institute, School of Health, Medical and Applied Sciences, Central Queensland University, Rockhampton, QLD 4702, Australia; m.j.hayman@cqu.edu.au

**Keywords:** pregnancy, physical activity, sedentary behavior, sleep

## Abstract

This study aimed to produce a list of the top 10 research priorities regarding physical activity, sedentary behavior, and sleep in pregnancy. Using the Delphi methodology, pregnant/postpartum individuals (*N* = 118), exercise professionals and healthcare providers (*N* = 55) listed up to 10 questions perceived as unanswered regarding physical activity, sedentary behavior, and sleep in pregnancy (Round 1). Respondents rated the proposed questions on a Likert importance scale (Round 2), and the sum of ratings received were totaled. Questions of priority regarding physical activity among pregnant/postpartum individuals (*N* = 67), healthcare providers and exercise professionals (*N* = 22) pertained to *exercise prescription, impact of exercise on maternal and fetal outcomes and impact of exercise on pregnancy conditions, special population groups* and *clinical education and access to information.* Sedentary behavior priorities included the *impact of sedentary behavior on maternal and fetal outcomes*, *sedentary recommendations* and *exercise and sedentary positioning*. Sleep research priorities included the *impact of pregnancy on sleep, safety, sleeping aids* and the effect of *exercise on sleep.* Pregnant/postpartum women, healthcare providers and exercise professionals prioritized questions that have in part been addressed by existing research, highlighting a need for improved knowledge translation from research to practice. They have also identified novel questions that warrant prioritization within future research.

## 1. Introduction

Physical activity guidelines for pregnant women have evolved significantly over the past 30 years from a recommended 1 mile of walking per day [1,2], to 150 minutes of moderate intensity physical activity each week [3,4,5,6,7]. Prenatal physical activity is associated with maternal health benefits, including a decreased risk of delivery by caesarean section, urinary incontinence, depressive symptoms, lumbopelvic pain and total gestational weight gain alongside reduced risk of developing gestational diabetes and pre-eclampsia [3,8]. It has also been deemed safe for the growing fetus, and reduces the risk of large for gestational age incidence [9,10].

Despite the provision of readily available prenatal physical activity guidelines and their associated health benefits, self-report data indicate that only 15% of pregnant individuals meet prenatal physical activity recommendations [11]. The barriers to physical activity are similar between pregnant and non-pregnant individuals (i.e., time, motivations, and social support); however, during pregnancy, they extend to physical limitations (including joint pain, swelling, back pain, leg cramps and discomfort), fatigue and a lack of knowledge regarding the types and volumes of exercise that are safe for the baby [12,13]. To date, it is unclear if research has overlooked, or perhaps not considered, specific aspects of prenatal physical activity that could help individuals overcome the physical and knowledge barriers that arise in pregnancy. It is therefore necessary to identify prenatal physical activity research needs among pregnant individuals and those involved in their care and management, to improve prenatal physical activity levels that could be of clinical significance for mum and baby. 

Further to physical activity, other modifiable lifestyle factors, such as sedentary behavior and sleep, have emerged within the non-pregnant literature and may be important considerations regarding maternal and fetal health. For example, sedentary behavior, defined as any waking behavior characterized by sitting, lying or in a reclining posture [14], is an independent cardiovascular and diabetes risk factor in non-pregnant populations [15]. In pregnancy, as physical activity declines with gestational age, sedentary behavior increases due to pregnancy-related fatigue and discomfort [16,17]. Sedentary behaviors are reportedly associated with adverse maternal cardiometabolic health and the delivery of macrosomic (large for gestational age) infants according to a systematic review [18]. Cross-sectional, objective evidence demonstrated that the pattern of these behaviors is also important; prolonged bouts of sedentary behavior, and not total sedentary time, is more closely associated with the development of gestational diabetes [19]. Despite emerging evidence with more than 25 studies reporting sedentary behaviors and maternal health outcomes [18], many of the studies are limited in their methodology by not conforming to gold-standard approaches of quantifying sedentary behavior [20]. Therefore, direction is necessary as to what questions prospective rigorous methodologies should focus on to advance this field of literature. 

In non-pregnant cohorts, poor sleep is inversely related to cardiovascular disease risk [21]. Although largely based on subjective data, women report poor sleep duration and/or quality in pregnancy [22,23,24]. Strikingly, Mindell et al. (2015) reported that 100% of their pregnant cohort (*n* = 2427) experienced disrupted sleep [24]. Furthermore, for some women, pre-existing sleep disorders may become exacerbated by pregnancy [25]. This is concerning since altered sleep in pregnancy is associated with adverse maternal (i.e., an elevated risk of developing gestational diabetes, hypertension and delivery by caesarean section) and fetal (i.e., preterm birth and fetal growth restriction) outcomes [25,26]. While non-pharmacological interventions can help improve sleep in pregnancy, including sleep hygiene considerations, education and cognitive behavioral therapy [27], there is a need to identify the specific research priorities of pregnant individuals and healthcare providers so that our understanding of this area can be developed [28]. This is important because to date, much of the literature regarding sleep in pregnancy is made up of subjective data, and thus is suboptimal in its design. By adopting a participant-centered approach, we can generate a novel hypothesis that will likely require objective data from adequately powered study samples to be accepted or rejected. Taken together, despite the paucity of evidence regarding sedentary behavior and sleep during pregnancy, both modifiable lifestyle factors likely contribute to maternal and fetal health; however, more research is needed to confirm this. As this field of literature expands, it seems timely to identify research priorities so that prospective evidence is both participant lead and clinically relevant, to yield maternal and fetal health-related impacts. 

Identifying research priorities can be achieved with the Delphi method—an approach that has been applied extensively within the literature and across a broad range of topics [29,30,31]. The Delphi method is a formal consensus technique used to obtain and evaluate the views of an expert group with knowledge and experience in a specialised area. The method involves the organization of an initial survey distributed to a population of interest that asks an open-ended question. The initial survey responses are collated by the research team, or steering panel, before redistribution to participants to achieve a high level of agreement across the hierarchy of responses (i.e., the responses are scored on a Likert scale of importance from 1–5). In classic Delphi studies, these results are then redistributed to evaluate the proportion of agreement between respondents until a consensus is reached about the level of agreement from participants on the scoring of the issues raised and typically involves a total of four rounds [30,31,32]. The Delphi method is flexible, and the number of rounds used differs from two, to typically four rounds depending on the research question, making it a useful and applied research tool [30,31,32,33,34]. As such, the Delphi method may not always strive to reach a consensus, rather it can be used to generate priorities in fields of research and/or practice.

The aim of this study was to adopt a modified Delphi method to produce a top 10 list of research priorities in pregnancy regarding physical activity, sedentary behavior and sleep among pregnant/postpartum individuals, healthcare providers and exercise professionals. This may help to direct future research to inform practice, and potentially contribute to the development of lifestyle-related guidelines that encompass physical activity, sedentary behavior and sleep recommendations for pregnant individuals.

## 2. Methods

### 2.1. Study Design

Between 11th March and 2nd June 2021, we recruited pregnant/postpartum individuals, defined as individuals who were pregnant or had delivered in the past 12 months, and prenatal healthcare providers and qualified exercise professionals, which included obstetric healthcare providers and exercise professionals (midwives, practitioners, physiotherapists, exercise physiologists, coaches and fitness instructors) who were working with pregnant individuals at the time of this study. The pregnant/postpartum cohort were recruited to gain their perspectives on the research needs they may have identified while pregnant. The healthcare and exercise professionals were recruited to provide a perspective on research needs they may have encountered while working with pregnant women. These groups were targeted since both work cohesively together throughout pregnancy, with the healthcare provider and exercise professional often being the primary source of information for a pregnant individual. By including both groups, we can determine what aspects of exercise, sleep and sedentary behavior are viewed as a research priority.

**Figure 1:**
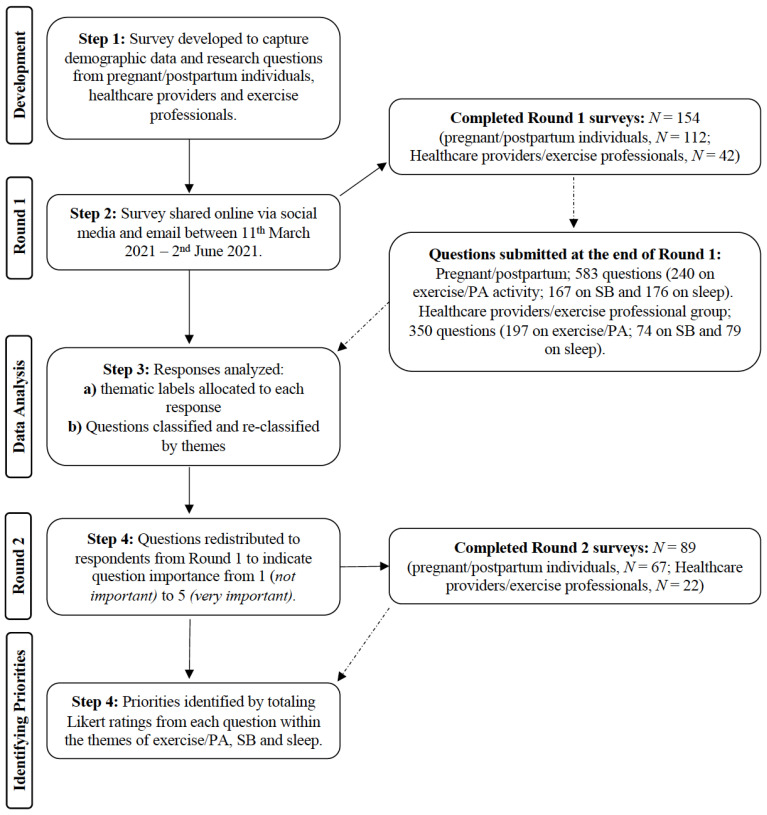
Modified Delphi approach used. PA, physical activity; SB, sedentary behavior.

These individuals (pregnant/postpartum, healthcare providers and exercise professionals) participated in a modified Delphi study described below and illustrated in Figure 1. We followed existing Delphi recruitment guidance to identify participants through passive and active recruitment processes [33,35]. The passive recruitment process involved advertising the study on social media and sending interested participants a link to the survey. The active recruitment process involved identifying key stakeholder groups, organizations and disciplines that were perceived to be key in identifying individuals that worked with pregnant women, including, clinicians and exercise professionals. A link to the survey was shared via email to academic and clinical networks, who were asked, if willing, if the survey could be forwarded to extended contacts with the expectation that this snowballing approach would further facilitate recruitment. Prior to completing the Delphi questionnaire, all potential participants were provided with an information sheet detailing the study. Potential participants were then asked to provide digital informed consent and an email address, all of which were collected and managed using the REDCap electronic data capture tools [36]. Only those that provided consent and an email address were included in the Round 1 analysis and sent the Round 2 survey.

### 2.2. The Delphi Method

In this study, the modified Delphi method comprised two rounds, both of which began with a sentence describing the purpose of the overall study and the respective round. Round 1 gathered basic demographic data and aimed to generate initial questions that pregnant/postpartum individuals, healthcare providers and exercise professionals viewed as unanswered relating to exercise/physical activity, sedentary behavior and sleep in pregnancy. This was achieved by asking pregnant/postpartum individuals to “list up to 10 questions that remain unanswered regarding exercise/physical activity, sedentary behavior and sleep during pregnancy respectively”. The healthcare providers and exercise professionals were asked to “think about their clinical or professional practice and list up to 10 research questions that remain unanswered regarding exercise/physical activity, sedentary behavior and sleep respectively during pregnancy”. The healthcare providers and exercise professionals were also asked if they discussed exercise/physical activity, sedentary behavior and sleep with pregnant individuals. The responses were subjected to content analysis to identify research priorities indicated by pregnant/postpartum individuals, healthcare providers and exercise professionals. Authors ÁB and MHD independently analyzed each response item by allocating a thematic label to each line, and then sorting the items by their given label. The authors then met to discuss and compare their labels until an agreement was reached. This approach was applied to generate the questions that would be redistributed for Round 2. Thematic labels and corresponding data were checked by MJH to ensure that they were representative of all responses.

Given the large volume of responses from Round 1, the questions were classified, and re-classified questions into comprehensive higher order themes until a consensus was achieved among the authors. This is in line with guidelines for the Delphi method to ensure that Round 2 would be manageable and not dissuade participation by presenting an onerous, repetitive and time-consuming survey [37]. This data processing has been applied elsewhere [32]. In line with the basic tenets of the Delphi technique, nothing was added or omitted from Round 2 unless the questions proposed were unrelated or irrelevant (i.e., pertained solely to the postpartum period) to the categories of interest [37].

Round 2 of the survey was distributed to all participants who consented to and completed Round 1. Round 2 presented the questions submitted in Round 1, which were organised into categories of physical activity, sedentary behavior and sleep. Participants were asked to indicate how important each of the questions generated from Round I were to them using a Likert scale ranging from 1 (*not important)* to 5 *(very important).* Participants were asked to answer with their first instinct to avoid deliberation. Pregnant/postpartum individuals were presented with 44 questions about exercise/physical activity, 21 about sedentary behavior and 22 about sleep, totaling 87 questions in Round 2. Obstetric healthcare providers and exercise professionals were presented with 25 questions about exercise/physical activity, 10 about sedentary behavior and 21 about sleep, totaling 56 questions in Round 2. The sum of ratings received for each question (on the Likert scale of 1–5) were then calculated to generate a total score [32].

### 2.3. Sampling Method

Similar to other Delphi studies, we did not base the sample size on achieving statistical power; rather, we aimed to recruit a diverse group of participants representative of a variety of experts relevant to the field, which included pregnant/postpartum individuals, healthcare providers and exercise professionals [38]. The literature proposes that a panel of 8–10 experts is sufficient to generate a range of opinions [33,35], and some studies have exceeded this [30,31]. To encourage diversity in our sample, the research study was advertised via social media platforms including Facebook, Instagram, Twitter and Reddit, with an expansive inclusion criterion (i.e., pregnant or postpartum within the past 12 months and/or experience working in pregnancy healthcare and/or exercise training) to all global regions. We also sent emails to healthcare providers and exercise professionals with reach among diverse groups. Furthermore, the survey was written at a level that allowed for those with basic English language skills to participate.

## 3. Results

A total of 118 pregnant/postpartum individuals and 55 healthcare providers/exercise professionals responded to Round 1, with six and thirteen of the surveys returned incomplete, respectively. Surveys were deemed incomplete if respondents did not provide an email address. Characteristics of the pregnant/postpartum group are presented in Supplemental Digital Content 1. In brief, 48% were pregnant (*N* = 54), and predominantly identified as white/Caucasian (*N* = 104). Respondents were mostly from Canada (*N* = 32), the United Kingdom (*N* = 30), Ireland (*N* = 18) and Australia (*N* = 11). The pregnant/postpartum group of participants submitted a total of 583 questions in Round 1 (240 on exercise/physical activity, 167 on sedentary behavior and 176 on sleep). The healthcare providers/exercise professional group of participants comprised exercise physiologists/kinesiologists, physiotherapists/physical therapists (*N* = 20), exercise practitioners (coach or fitness instructor) (*N* = 10), medical practitioners (physician, registered nurse or midwife) (*N* = 10), a dietician (*N* = 1) and a radiographer (*N* = 1). The healthcare providers/exercise professionals group of participants submitted a total of 350 questions (197 on exercise/physical activity, 74 on sedentary behavior and 79 on sleep). The majority of health care providers and exercise professionals reported that they discussed exercise/physical activity (88%), sedentary behavior (70%) and sleep (81%) with pregnant individuals. This group (the healthcare providers and exercise professionals) reported working with pregnant individuals for 8 ± 6 years.

Following content analysis, a final list of 87 questions was presented to pregnant/postpartum individuals (*n* = 112) and 56 to healthcare providers/exercise professionals (*n* = 42) for ranking in Round 2. Then 60%(*n* = 67) of pregnant/postpartum individuals and 52% (*n* = 22) healthcare providers and exercise professionals ranked the questions presented to them in Round 2 in terms of their importance to be researched. All responses to Round 2 were sorted within their respective category of exercise/physical activity, sedentary behavior and sleep. Responses were then sorted by the total score to produce a list of the top 10 research questions proposed by pregnant/postpartum individuals, healthcare providers and exercise professionals for exercise/physical activity, sedentary behavior and sleep are listed in Table 1, Table 2 and Table 3, respectively. The top 10 research priorities regarding physical activity among the pregnant/postpartum group for physical activity pertained to themes *exercise prescription, impact of exercise on maternal and fetal outcomes and impact of exercise on pregnancy conditions.* This was similar for healthcare providers and exercise professionals with the addition of questions relating to *special population groups,* and *clinical education and access to information* (Table 1). Regarding sedentary behavior, the top 10 research priorities among pregnant/postpartum individuals were concerned about the *impact of sedentary behavior on maternal and fetal outcomes*, *sedentary recommendations* (i.e., safe durations of being sedentary) and *exercise and sedentary positioning* (i.e., exercise to help with posture and pain when sitting). Healthcare providers and exercise professionals prioritized similar topics; however, questions pertaining to *sedentary positioning* were about safe positions for mum and baby (Table 2). For sleep, pregnant/postpartum individuals prioritized questions that related to themes of *the impact of pregnancy on sleep, safety, sleeping aids* and the role of *exercise.* This was similar for the healthcare provider and exercise professionals—they prioritized questions about the *impact of pregnancy on sleep, maternal sleep and pregnancy outcomes/complications, exercise and sleep and sleep aids* (Table 3).

## 4. Discussion

This modified Delphi study produced a top 10 list of research questions during pregnancy regarding physical activity, sedentary behavior and sleep according to pregnant/postpartum individuals, healthcare providers and exercise professionals. To the best of our knowledge, this has been the only attempt to engage a lay and clinical/professional group to identify research priorities within these areas (physical activity, sedentary behavior and sleep). These identified research priority lists may help to direct future research and potentially assist funding bodies in developing research agendas and funding calls. The lists have also identified a need for knowledge translation from existing research to pregnant individuals, healthcare providers and exercise professionals. As such, this study has exposed a great need for improving research dissemination to improve the information needs of the groups included.

### 4.1. Exercise/Physical Activity

A number of the top 10 questions proposed by pregnant/postpartum individuals about physical activity have been addressed by the most recent prenatal physical activity guidelines [3,4,5,6,7]. This highlights the critical lack of knowledge translation between the literature and related practices. While it would seem pertinent that this translation could arise at the healthcare provider and exercise professional level, this group also presented uncertainty regarding the prescription, safety and benefits of exercise in pregnancy, all of which have been alluded to in recent prenatal physical activity guidelines [3,4,5,6,7]. According to our data and supported by others, healthcare providers may not always be familiar with current prenatal physical activity recommendations [39,40]. This may be due to a lack of training and/or awareness to support physical activity prescription for clinical populations [41]. It is plausible that until this is addressed, we may continue to observe a shortfall in the number of pregnant individuals receiving prenatal physical activity recommendations from their healthcare providers and/or exercise professionals. Critically, both groups identified a need to know more about physical activity with pregnancy complications, and while this area has grown, it warrants further expansion within the literature [42].

Other questions within the physical activity domain are novel and have yet to be addressed by prenatal physical activity research. For example, the influence of exercise on long-term maternal and fetal outcomes remain largely unexplored. According to animal models, prenatal exercise has a positive influence on offspring cardiometabolic health and physical developmental [43]; however, few studies include long-term follow up of the health and development of the offspring, especially beyond the first few months of life [9]. Another important question raised relates to the maternal and fetal responses when physical activity levels are substantially exceeded. The longer the duration and/or volume of physical activity, the greater the reduction in circulating blood glucose (acutely), and the greater the reduction in pregnancy complications such as gestational diabetes, preeclampsia and gestational hypertension when performed habitually [8]. Nevertheless, more work is needed to understand how these and other markers of maternal health, such as blood pressure and vascular responses, alongside fetal health respond to acute and chronic exposure to long durations of physical activity.

Lastly, the pregnant/postpartum participant group identified the need to understand more about the longer-term influence of prenatal physical activity, specifically as it related to recovery following childbirth. Future research that can allude to the influence of prenatal physical activity on postpartum recovery is, therefore, important to this group.

### 4.2. Sedentary Behavior

According to the World Health Organization, the guidelines regarding sedentary behavior in pregnancy can be extrapolated from non-pregnant populations due to the lack of evidence available [44]. Sedentary behavior is an emerging cardiovascular risk factor in non-pregnant populations [45]; however, our knowledge to date regarding its role in pregnancy is limited. Thus far, sedentary behavior has been shown to be to associated with adverse maternal and fetal outcomes [18], which include preterm delivery and inhibited fetal growth [17]. It is, therefore, encouraging that both participant groups have prioritized the need for the research to further investigate the impact of sedentary behavior on the immediate and long-term health of both mother and baby. In non-pregnant populations, prolonged sedentary behavior leads to reduced cerebral blood flow and vascular dysfunction [46,47]—both of which may be detrimental to cerebrovascular, cardiovascular and metabolic health. Encouragingly, however, these detriments to cardiovascular dysfunction can be attenuated, at least in non-pregnant individuals, by breaking up long bouts of sedentary time with short (two-minute) walking breaks; this has yet to be assessed in pregnancy [47]. Thus, identifying thresholds of sedentary time and pregnancy outcomes is warranted, along with potential therapies if linked with adverse outcomes.

### 4.3. Sleep

Compared to the use of accelerometry in pregnancy to quantify physical activity, there is an even greater paucity of evidence quantifying sleep in pregnancy by objective means. This is reflected by the diverse and novel questions proposed and prioritized by the groups in this study. The themes identified within the sleep category were similar between both groups; however, the importance placed on the themes differed. For the pregnant/postpartum group, identifying safe sleep position(s) was of paramount importance, followed by evidence-based strategies to improve sleep. Furthermore, pregnant/postpartum individuals want to know more about the impact of pregnancy on sleep including the types and durations of sleep that are atypical in pregnancy, the causes of poor/disrupted sleep/insomnia and the difference between pregnant and non-pregnancy sleep-wake cycle.

Healthcare providers and exercise professionals highlighted the need for more research on the impact of optimal and suboptimal sleep quality on pregnancy outcomes, mental health and fatigue. They also identified a need to identify interventions to improve sleep quality during pregnancy. Similar to the pregnant/postpartum group, questions pertaining to safe sleeping positions, atypical sleeping patterns and the interrelationship between exercise and sleep were also prioritized within the top 10 research questions. It is apparent that this area of research is in its infancy and warrants objective experimental study designs to begin to address the proposed questions using objective data collection methods [48,49,50].

A noteworthy observation with the proposed questions from the modified Delphi study includes the concern among both groups about the impact of one lifestyle behavior over another. For example, pregnant/postpartum individuals wanted to know about how much they should break up their sedentary behavior (i.e., with physical activity), as well as the influence of physical activity on sleep. Similarly, healthcare providers and exercise professionals wanted to know about what exercises individuals could do to alleviate back and pelvic pain associated with sedentary behavior, and about the relationship between sleep and exercise habits. As highlighted by the Australian guidelines for prenatal physical activity [7], there are plausible interactions between exercise and sleep; however, the lack of evidence to date does not allow for any robust inference to be made. According to Whitaker et al. (2021), physical activity, sedentary behavior and sleep are modified in some way in pregnancy and plausibly may interact with one another. Interestingly, the authors found that individuals with less optimal sleep patterns engaged with low levels of sedentary behavior, which may be lifestyle (stress and responsibilities) and not pregnancy related [51]. On the other hand, poor sleep quality is associated with low levels of physical activity, suggesting that the relationship between sleep and activity levels is bidirectional. This presents rationale to consider each of these factors collectively to help manage and optimize prenatal health. They are clearly of interest to pregnant/postpartum individuals, healthcare providers and exercise professionals and, therefore, warrant consideration in future studies to develop our understanding about how they interact with one another.

## 5. Strengths and Limitations

This study is the first to identify research priorities among pregnant/postpartum individuals, healthcare providers and exercise professionals to guide future exercise/physical activity, sedentary behavior and sleep research during pregnancy. Secondary to this, we identified information needs among these cohorts and thus a clear demand for research dissemination. There are notable strengths of this study. For instance, the study drew upon currently pregnant and recently postpartum individuals from around the world, and thus the proposed questions represent current and global knowledge gaps regarding physical activity, sedentary behavior and sleep in pregnancy. The healthcare providers and exercise professional group were all currently working with pregnant individuals and are, therefore, again representative of the current knowledge gaps. This study is therefore, stakeholder driven and has deviated for the first time in this field, from generating a researcher-driven hypothesis. Another notable strength of this study is the use of the modified Delphi method, which is accessible, of low participant burden, and gathers repeated input from respondents. This likely contributed to our ability to exceed a panel of 10–18 experts recommended elsewhere to generate a range of opinions [33,35]. Lastly, the sample responses came from Canada, the United Kingdom, Ireland, Australia and the United States. Since physical activity guidelines are similar in pregnancy between countries, the data presented a homogenous representation of research priorities in this field. 

This study is not without its limitations Firstly, with this type of study, there may be an assumption that pregnant individuals, healthcare providers and exercise professionals are up to date with the current literature. According to our findings, this was not always the case, and perhaps a stricter inclusion criterion could have yielded a different outcome. Secondly, the response rate is similar to previously published Delphi studies [29,52], although falls short of the recommended 70% for Round 2 of the survey [37]. Additionally, while the sample is predominantly derived from five developed westernized countries, the findings are, therefore, not representative of non-western countries. It is likely that research priorities and information needs would differ in countries where healthcare systems are challenged. Lastly, it is plausible that the sample of respondents had an invested interest in understanding more about physical activity, sedentary behavior and/or sleep in pregnancy, or in practice and, therefore, the proposed questions may not be generalizable to all pregnant individuals, healthcare providers and exercise professionals.

## 6. Conclusions

To conclude, this study produced a top 10 list of priority research questions regarding exercise/physical activity, sedentary behavior and sleep according to pregnant/postpartum individuals, healthcare providers and exercise professionals. We also indirectly identified the information needs of pregnant/postpartum individuals, healthcare providers and exercise professionals regarding exercise/physical activity, sedentary behavior and sleep. The exercise/physical activity category contained questions that have been addressed by the most up to date guidelines and, as such, highlighted a gap in the knowledge translation from the literature to lay and professional groups. The sedentary behavior and sleep categories showed consistent priorities between pregnant/postpartum individuals, and healthcare providers and exercise professionals. Indeed, while future research may target these proposed questions, it is pertinent that the knowledge translation of evidence to practice also takes place if we are going to truly improve the health and wellbeing of pregnant individuals and their progeny.

## Figures and Tables

**Table 1:** Top 10 exercise research priorities by theme according to pregnant/postpartum women, healthcare providers and exercise professionals (for the full list of questions and scores, see Supplemental Digital Content 2).

Rank	Exercise/Physical Activity Research Question	Total
**Pregnant/Postpartum Group**		
	**Exercise Prescription**	
1	What types of exercises should I avoid because they are unsafe?	313
2	What modifications should I make to my exercise as my pregnancy progresses?	298
8	What exercise prescription should I use to guide my pelvic floor exercises-sets, repetitions, how long should each contraction hold for etc?	285
9	What impact does exceeding exercise guidelines have on maternal and fetal outcomes?	284
10	What intensity should I exercise at?	282
	**Impact of exercise on maternal and fetal outcomes**	
5	What are the short and long-term maternal and fetal benefits to exercise in pregnancy?	287
	**Impact of exercise on pregnancy conditions**	
3	What impact does exercise have on recovery?	290
4	What impact does exercise have on labour?	289
6	What impact does exercise have on birthing outcomes?	287
7	What impact does exercise have on pregnancy related complications (i.e., gestational diabetes, pre-eclampsia, hyperemesis, insomnia, risk of miscarriage)?	286
**Healthcare providers and Exercise Professional Group**		
	**Exercise Prescription**	
1	What exercise is recommended for pregnant women with complications? (i.e., gestational diabetes, placenta previa, diastis recti, pubic symphysis, advanced maternal age, depression)	110
2	What type (i.e., aerobic, weights, inversions) and intensities (light, moderate, high) of exercise are safe and beneficial for an active and inactive pregnant woman?	103
4	What are the contraindications to exercise in pregnancy?	100
7	How much exercise should a pregnant woman engage with, and does it change across the gestation?	95
	**Impact of exercise on maternal and fetal outcomes**	
5	What short and long-term benefits are associated with exercise during pregnancy for mother and baby? (i.e., complications, labour, immunity, weight gain)	99
	**Impact of exercise on pregnancy conditions**	
3	What impact does exercise (aerobic and resistance training) have on pelvic floor health?	102
8	What is the association between physical activity level and incontinence?	94
6	How can women safely exercise with a prolapse?	99
	**Special populations Groups**	
9	What exercise recommendations are in place for pregnant athletes?	94
	**Clinical education and access to information**	
10	Do pregnant women seek/receive information about exercise and are healthcare providers educated about exercise in pregnancy?	94

**Table 2:** Top 10 sedentary behavior research priorities by theme according to pregnant/postpartum women, healthcare providers and exercise professionals (for full overall league table, see Supplemental Digital Content 3).

Rank	Sedentary Behavior Research Question	Total
**Pregnant/Postpartum Group**		
	**Impact of SB on maternal and fetal health outcomes**	
1	How does SB impact labour (i.e., type, timing, duration, intervention)?	294
2	Does SB influence risk of pregnancy related complications? (i.e., gestational diabetes, pre-eclampsia, morning sickness, weight gain, deep vein thrombosis, insomnia).	286
3	How does SB impact the immediate and long-term health for mum and baby?	283
4	How does SB impact the physiological changes in pregnancy? (i.e., increased heart rate, blood volume, cardiac output etc.)	275
5	Do pregnancy outcomes (baby size, mother’s weight gain, health problems for mom or baby, etc) differ between those that engage with sedentary work compared with those who work 8 hours on their feet?	273
6	Is SB associated with increased pelvic and lower back pain, stiffness, fatigue, and muscle weakness?	272
10	Do pregnancy outcomes differ between individuals with obesity who are active compared to those who are not?	258
	** SB Recommendations **	
7	How much SB is too much and does this change over the pregnancy duration?	269
8	How long is safe to be sedentary for and/or how often should I break up my SB?	264
	**Exercise and Sedentary Positioning**	
9	What exercises should pregnant women do to improve sitting posture/reduce back pain perhaps?	263
**Healthcare providers and Exercise Professional Group**		
	**Impact of SB on maternal and fetal health outcomes**	
1	What is the immediate and long-term impact of SB on mother and baby health?	98
2	What risks and complications are associated with SB in pregnancy? (i.e., birth experience, mode & length of labour)	97
4	How does SB impact joint and pelvic pain in pregnant women classed with normal weight or obesity?	93
5	Are mums made aware of the risks associated with SB in pregnancy?	92
6	What impact does SB have on pelvic floor including pelvic tilt?	87
	**Sedentary positioning**	
10	What sedentary positions are safe/optimal in pregnancy and does this change across trimesters?	78
	**SB Recommendations**	
3	Are healthcare providers discussing SB with pregnant women?	94
7	What are the barriers to limiting SB in pregnancy?	84
8	What is the optimal length of time that pregnant women should be sedentary for?	81
	**Other**	
9	Why are some pregnant women more sedentary than others?	80

SB; sedentary behavior.

**Table 3:** Top 10 sleep research priorities by theme according to pregnant/postpartum women (for full overall league table, see Supplemental Digital Content 4).

Rank	Sleep Research Question	Total
**Pregnant/Postpartum Group**		
	**Impact of pregnancy on sleep**	
4	What causes poor sleep quality in pregnancy?	266
5	What type and duration of sleep is atypical in pregnancy and does this differ across trimesters?	263
6	Why do pregnant women have insomnia and what can be done to help with this?	263
7	What is the function of regular waking in pregnancy and how does this impact maternal sleep?	262
9	Is circadian rhythm (sleep/wake cycle) different in pregnant versus non-pregnant?	255
	**Safety**	
1	What is the best/safest position for sleep during pregnancy for both maternal and fetal health? (i.e., back, belly, right/left side)	286
2	What sleeping positions are best to avoid/protect back, neck and hip pain, cramp and pins and needles?	283
	**Sleeping Aids**	
3	How can I improve my sleep (naturally and otherwise) in pregnancy?	276
	**Exercise and Sleep**	
8	How important is it to rest if tired, should I push through and do some exercise instead?	262
10	Are there any specific exercise considerations to improving sleep?	253
**Healthcare providers and Exercise Professional Group**		
	**Impact of pregnancy on sleep**	
2	How can pregnant women improve sleep quality? (i.e., exercise)	99
3	How does sleep quality impact maternal mental health? (Including bonding with baby, forgetfulness)	98
7	Is there an association between sleep and energy levels in pregnancy?	89
10	What are the causes and implications of pregnancy related insomnia?	86
6	What sleep patterns (duration, quality, stages) are typical in pregnancy and does this change across trimesters?	90
	**Maternal sleep and pregnancy outcomes/complications**	
9	What sleep positions are best in pregnancy-are some linked to pregnancy related complications?	88
1	What are the benefits/implications of sleeping enough/less than recommended? (i.e., on pregnancy outcomes)	100
4	How does maternal sleep impact immediate and long term fetal health?	96
	**Exercise and Sleep**	
5	Is there a link between stress, lack of sleep and poor exercise habits?	90
	**Sleeping Aids**	
8	What pharmacotherapy is beneficial and safe to improve sleep in pregnancy? (i.e., melatonin)	88

## Data Availability

Data are contained within the article and within Supplementary Materials.

## Supplementary Materials

The following supporting information can be downloaded at: https://www.mdpi.com/article/10.3390/ijerph19052909/s1, Supplemental Digital Content 1—Pregnant and Postpartum Characteristics. Supplemental Digital Content 2—Exercise Questions. Supplemental Digital Content 3—Sedentary Behavior Questions. Supplemental Digital Content 4—Sleep Questions.
