# Supplementary material for: A Delphi Study to Identify Research Priorities Regarding Physical Activity, Sedentary Behavior and Sleep in Pregnancy
Source: Int J Environ Res Public Health. 2022 Mar 2;19(5):2909. doi: 10.3390/ijerph19052909 (PMC8909963; doi:10.3390/ijerph19052909)
Supplement: Supplementary file 1 [file ijerph-19-02909-s001.zip › Supplemental Digital Content 1 - Pregnancy and Postpartum Characteristics.pdf]

Participant Characteristics for the pregnant/postpartum group

	Number (% out of 112)
Pregnant	54 (48%)
**Ethnic Background**	
White/Caucasian	104 (94%)
Mixed Heritage	3 (3%)
Asian	1 (1%)
American Indian, Alaska Native, First Nations, Inuit and Métis	1 (1%)
South Asian	1 (1%)
Other: Chinese	1 (1%)
**Country of Residence**	
Australia	11 (10%)
Canada	32 (39%)
Ireland	18 (16%)
New Zealand	1(1%)
Poland	1 (1%)
Singapore	1 (1%)
South Africa	1 (1%)
United Kingdom	30 (27%)
USA	4 (4%)
**Pregnancy Complications**	
Gestational diabetes	6 (6%)
Preeclampsia	1 (1%)
Eclampsia	2 (2%)
Placenta previa	2 (2%)
Pre-term labour	1 (1%)
Short cervix	2 (2%)
Depression	5 (5%)
Other (including iron deficiency, gestational hypertension, hypothyroidism, intrauterine growth restriction, anxiety)	22 (20%)
