# Supplementary material for: A Delphi Study to Identify Research Priorities Regarding Physical Activity, Sedentary Behavior and Sleep in Pregnancy
Source: Int J Environ Res Public Health. 2022 Mar 2;19(5):2909. doi: 10.3390/ijerph19052909 (PMC8909963; doi:10.3390/ijerph19052909)
Supplement: Supplementary file 1 [file ijerph-19-02909-s001.zip › Supplemental Digital Content 2 - Exercise Questions.pdf]

Exercise related factor according to pregnant/postpartum women	Total	Themes
What types of exercises should I avoid because they are unsafe?	313	1
What modifications should I make to my exercise as my pregnancy progresses?	298	1
What impact does exercise have on recovery?	290	3
What impact does exercise have on labour?	289	3
What are the short and long-term maternal and fetal benefits to exercise in pregnancy?	287	2
What impact does exercise have on birthing outcomes?	287	3
What impact does exercise have on pregnancy related complications (i.e., gestational diabetes, pre-eclampsia, hyperemesis, insomnia, risk of miscarriage)?	286	3
What exercise prescription should I use to guide my pelvic floor exercises - sets, repetitions, how long should each contraction hold for etc?	284	1
What impact does exceeding exercise guidelines have on maternal and fetal outcomes?	284	1
What intensity should I exercise at?	282	1
What are the maternal and fetal benefits to exercise?	280	2
What impact does exercise have on mental health (including stress, depression and anxiety)?	280	3
How do different types of exercise affect the pelvic floor (tighten and relax)?	280	5
Is there a minimum and maximum amount of exercise I should be doing in pregnancy?	276	1
How much exercise is safe?	274	1
How can I exercise safely with diastasis recti? (Partial or complete separation of the abdominal muscles)	268	5
What impact does exercise have on diastasis recti? (Partial or complete separation of the abdominal muscles)	267	3
Does pre-pregnancy fitness influence exercise benefits, risks, frequency and intensity (i.e. heart rate) during pregnancy?	265	6
What types of resistance training exercises should I be doing?	259	1
How should I measure exercise intensity?	258	1
How does my body change in pregnancy and how does this affect exercise during pregnancy?	256	4
How can I exercise safely with a prolapse?	256	5
How should nutritional intake change when you exercise during pregnancy?	255	8
What additional clinical and/or community-based services could be provided to help pregnant individuals?	252	8
What types of aerobic activity should I be doing?	251	1
How does pelvic girdle pain affect exercise?	248	5
What exercise prescription should I use to guide resistance training sessions - sets, reps, exercises, rest, intensity etc?	246	1
Does age influence exercise prescription?	244	6
What modifications/adaptations should be made in the workplace to accommodate pregnant individuals?	244	7
What can be done to encourage exercise participation and adherence in pregnancy?	244	8
Will my health care provider/professional provide guidance on exercise during pregnancy?	242	1
Why do some exercises cause Braxton hicks contractions?	241	8
What impact does exercise have on fertility and IVF?	239	3
Are there support clothing that could help with exercising with a bump especially later in pregnancy?	233	8
How do I commence an exercise program if I have been previously inactive?	230	6
Is it OK to commence exercise if previously inactive?	228	6
What impact does exercise have on body image?	226	3
Do athletes have increased prevalence of pregnancy complications and/or adverse outcomes?	225	6
Is it OK to lose weight during pregnancy?	216	8
How well do partners understand and/or become involved in supporting their partners need to exercise in pregnancy?	214	8
What exercises can I do while sitting at my desk or in meetings?	210	7
Can I expect to have sore muscles after exercising in pregnancy and if so, should I persevere?	204	8
What is the impact of gym and pool closures during the COVID-19 pandemic on pregnant individuals?	200	8
Are in person or virtual exercise programs better for adherence?	192	8

**Exercise Theme Key**	
**1**	Exercise Prescription
**2**	Impact of exercise on maternal and fetal outcomes
**3**	Impact of exercise on pregnancy conditions
**4**	Anatomical and Physiological Adaptations
**5**	Exercise and specific conditions
**6**	Special Populations Groups
**7**	Occupational exercise considerations
**8**	Other

Exercise related research questions according to healthcare providers and exercise professionals	Total	Themes
What exercise is recommended for pregnant women with complications? (i.e. gestational diabetes, placenta previa, diastis recti, pubic symphysis, advanced maternal age, depression)	110	5
What type (i.e., aerobic, weights, inversions) and intensities (light, moderate, high) of exercise are safe and beneficial for active and inactive pregnant women?	103	1
What impact does exercise (aerobic and resistance training) have on pelvic floor health?	102	3
What are the contraindications to exercise in pregnancy?	100	1
What short and long-term benefits are associated with exercise during pregnancy for mother and baby? (i.e. complications, labour, immunity, weight gain)	99	2
How can women safely exercise with a prolapse?	99	5
How much exercise should a pregnant woman engage with, and does it change across the gestation?	95	1
What is the association between physical activity level and incontinence?	94	3
What exercise recommendations are in place for pregnant athletes?	94	6
Do pregnant women seek/receive information about exercise and are healthcare providers educated about exercise in pregnancy?	94	7
What are the minimum requirements for exercise professionals to teach physical activity classes to pregnant women?	93	7
How can exercise in pregnancy help manage pain? (i.e., in labour, pelvic girdle, back)	92	3
Can exercise interventions improve fertility in women with obesity?	92	5
What is the influence of exercise on racial disparities/differences in pregnancy?	92	6
Can a publicly funded maternal health program focused on maternal health during pregnancy improve pregnancy outcomes (including maternity cost) and is it feasible?	91	7
What are the barriers to exercise/accessing appropriate information about exercise in pregnancy?	90	7
What are the guidelines regarding prone and supine exercise in pregnancy and does this change across trimesters?	88	1
Does use of a vaginal support pessary for exercise in pregnancy in women at higher risk of developing vaginal prolapse (e.g., connective tissue disorders, family history of prolapse) reduce the long-term risk of prolapse occurrence/severity?	88	5
Does heat stress and acute increases in core body temperature via sauna or hot tub cause a danger to the foetus?	88	1
How do pregnancy related physiological changes (i.e., ligament laxity, hormones, weight gain, balance) impact women's ability and motivation to exercise in pregnancy?	87	2
Is working with a qualified pregnancy-trained exercise professional more beneficial in pregnancy compared with exercising alone?	87	8
What are the nutrition and fluid needs for pregnant women aiming to build/maintain muscle mass?	86	8
How can pregnant women monitor exercise intensity and training load?	85	1
Do supportive garments help women to remain active in pregnancy?	75	8
Should a pregnant woman adjust their position on a bike to optimise comfort and limit any pressure on the fetus?	70	8

**Exercise Theme Key**	
1	Exercise prescription
2	Exercise on maternal and fetal outcomes
3	Impact of exercise on pregnancy conditions
4	Anatomical and Physiological Adaptations
5	Exercise and Specific Conditions
6	Special populations Groups
7	Clinical education and access to information
8	Other
