# Supplementary material for: A Delphi Study to Identify Research Priorities Regarding Physical Activity, Sedentary Behavior and Sleep in Pregnancy
Source: Int J Environ Res Public Health. 2022 Mar 2;19(5):2909. doi: 10.3390/ijerph19052909 (PMC8909963; doi:10.3390/ijerph19052909)
Supplement: Supplementary file 1 [file ijerph-19-02909-s001.zip › Supplemental Digital Content 3 - Sedentary Behaviour Questions.pdf]

SB related factor according to pregnant/postpartum women	Total	Theme
How does SB impact labour (i.e. type, timing, duration, intervention)?	294	1
Does SB influence risk of pregnancy related complications? (i.e. gestational diabetes, pre-eclampsia, morning sickness, weight gain, deep vein thrombosis, insomnia).	286	1
How does SB impact the immediate and long term health for mum and baby?	283	1
How does SB impact the physiological changes in pregnancy? (i.e. increased heart rate, blood volume, cardiac output etc.)	275	1
Do pregnancy outcomes (baby size, mother's weight gain, health problems for mom or baby, etc) differ between those that engage with sedentary work compared with those who work 8 hours on their feet?	273	1
Is SB associated with increased pelvic and lower back pain, stiffness, fatigue and muscle weakness?	272	1
How much SB is too much and does this change over the pregnancy duration?	269	2
How long is safe to be sedentary for and/or how often should I break up my sedentary behaviour?	264	2
What exercises should pregnant women do to improve sitting posture / reduce back pain perhaps?	263	3
Do pregnancy outcomes differ between individuals with obesity who are active compared to those who are not?	258	1
Does SB cause limb swelling and restless leg syndrome in pregnancy?	252	1
Does SB impact foetal position?	251	1
What sedentary position is safe for pregnant women and their baby? (i.e. seat height, legs open/crossed, recline vs upright vs lounging).	249	4
If pre-pregnancy physical activity levels have reduced and SB has increased, how does this impact baby compared with a pregnancy where mum was entirely sedentary pre-pregnancy?	245	3
Are sedentary positions dependent of trimester of pregnancy and/or when should they change? (i.e. ball versus chair)	244	4
Is there a difference between rest and SB and how can I rest without being too sedentary?	243	5
Is there a relationship between SB and IVF success rates?	237	6
How can we lessen mum's guilt about not exercising in pregnancy if they feel unwell?	235	3
Why is a sedentary lifestyle accepted as the norm in pregnancy?	224	6
Is there an optimal time of day where rest and recovery are encouraged?	223	2
Is it helpful to be on bedrest for safety of the baby?	213	2

SB; sedentary behaviour

**SB Theme Key**	
**1**	Impact of SB on maternal and fetal health outcomes
**2**	SB recommendations
**3**	Exercise and Sedentary Positioning
**4**	Sedentary Positioning
**5**	What is the difference between rest and SB?
**6**	Other

SB; sedentary behaviour

**SB related factor according to healthcare providers and exercise professionals**	Total	Theme
What is the immediate and long-term impact of SB on mother and baby health?	98	1
What risks and complications are associated with SB in pregnancy? (i.e. birth experience, mode & length of labour)	97	1
Are healthcare providers discussing SB with pregnant women?	94	3
How does SB impact joint and pelvic pain in pregnant women classed with normal weight or obesity?	93	1
Are mums made aware of the risks associated with SB in pregnancy?	92	1
What impact does SB have on pelvic floor including pelvic tilt?	87	1
What are the barriers to limiting SB in pregnancy?	84	3
What is the optimal length of time that pregnant women should be sedentary for?	81	3
Why are some pregnant women more sedentary than others?	80	4
What sedentary positions are safe/optimal in pregnancy and does this change across trimesters?	78	2

**Sedentary Behaviour Theme Key**	
1	Impact of SB on maternal and fetal health outcomes
2	Sedentary positioning
3	SB recommendations
4	Other
