# Supplementary material for: A Delphi Study to Identify Research Priorities Regarding Physical Activity, Sedentary Behavior and Sleep in Pregnancy
Source: Int J Environ Res Public Health. 2022 Mar 2;19(5):2909. doi: 10.3390/ijerph19052909 (PMC8909963; doi:10.3390/ijerph19052909)
Supplement: Supplementary file 1 [file ijerph-19-02909-s001.zip › Supplemental Digital Content 4 - Sleep Questions.pdf]

Sleep Related Factor according to pregnant/postpartum women	Total	Theme
What is the best/safest position for sleep during pregnancy for both maternal and fetal health? (i.e. back, belly, right/left side)	286	2
What sleeping positions are best to avoid/protect back, neck and hip pain, cramp and pins and needles?	283	2
How can I improve my sleep (naturally and otherwise) in pregnancy?	276	3
What causes poor sleep quality in pregnancy?	266	1
What type and duration of sleep is atypical in pregnancy and does this differ across trimesters?	263	1
Why do pregnant women have insomnia and what can be done to help with this?	263	1
What is the function of regular waking in pregnancy and how does this impact maternal sleep?	262	4
How important is it to rest if tired, should I push through and do some exercise instead?	262	4
Is circadian rhythm (sleep/wake cycle) different in pregnant versus non-pregnant?	255	1
Are there any specific exercise considerations to improving sleep?	253	4
Is there an interaction between sleep and pregnancy related hormones?	250	1
How does bladder heaviness impact sleep quality?	246	1
What impact does sleep deprivation have on exercise performance and recovery?	240	4
Is there an association between sleep quality/quantity and pelvic pain?	236	1
Should I take naps during the day and how do they affect mum and baby?	232	1
Does the developing baby have a circadian rhythm (sleep/wake cycle)?	224	1
What is the effectiveness of sleeping aids (i.e. supplements, weighted blankets)?	208	3
Why do pregnant women have vivid dreams?	199	1
Why is baby more active when I am trying to sleep?	195	1
Can I overheat while sleeping and harm my baby?	193	2
How does changing time zones and sleep patterns affect baby health?	192	6
Do black out blinds/eye mask influence pregnancy related sleep?	180	3

	Sleep Theme Key
**1**	Impact of pregnancy on sleep
**2**	Safety
**3**	Sleeping Aids
**4**	Exercise and sleep
**5**	Other

Sleep related factor according to healthcare providers and exercise professionals	Total	Theme
What are the benefits/implications of sleeping enough/less than recommended? (i.e., on pregnancy outcomes)	99	3
How can pregnant women improve sleep quality? (i.e., exercise)	99	1
How does sleep quality impact maternal mental health? (Including bonding with baby, forgetfulness)	98	1
How does maternal sleep impact immediate and long term fetal health?	96	3
Is there a link between stress, lack of sleep and poor exercise habits?	90	4
What sleep patterns (duration, quality, stages) are typical in pregnancy and does this change across trimesters?	89	2
Is there an association between sleep and energy levels in pregnancy?	89	1
What pharmacotherapy is beneficial and safe to improve sleep in pregnancy? (i.e., melatonin)	88	5
What sleep positions are best in pregnancy - are some linked to pregnancy related complications?	88	3
What are the causes and implications of pregnancy related insomnia?	86	1
Could a pelvic floor training program in pregnancy reduce bladder pressure and therefore sleep disturbance?	86	4
How does sleep during pregnancy relate to physiological changes in neural networking and adaptations?	86	1
Do sleep hygiene recommendations differ between pregnant and non-pregnant state?	86	2
Are there any clinical tools to predict sleep apnoea in pregnancy?	85	3
What factors affect how much sleep women get in pregnancy/ postpartum period?	85	1
Does lack of sleep impact exercise adherence and tolerance during pregnancy?	84	4
Do healthcare professionals discuss sleep patterns and sleep hygiene with pregnant women?	83	2
What are the reasons for sleep disturbances in pregnancy?	81	1
Are mums aware of the importance of sleep in pregnancy?	79	3
How does sleep affect muscle soreness from strength training in pregnancy?	79	4
Is napping recommended?	77	6

	Sleep Theme Key
1	How is sleep impacted during pregnancy?
2	What does good sleep hygiene look like in pregnancy (i.e. pre bed time, food, caffeine)
3	Maternal sleep and pregnancy outcomes/complications
4	Exercise and Sleep
5	Sleeping Aids
6	Other
